# Heart Rate Variability Differences by Match Phase and Outcome in Elite Male Finnish Padel Players

**DOI:** 10.3390/jfmk10030306

**Published:** 2025-08-08

**Authors:** Rafael Conde-Ripoll, Antonin Jamotte, Jose A. Parraca, Álvaro Bustamante-Sánchez

**Affiliations:** 1 Department of Sports Sciences, Faculty of Medicine, Health and Sports, Universidad Europea de Madrid, C/Tajo, s/n, 28670 Villaviciosa de Odón, Madrid, Spain; info.conderipoll@gmail.com; 2 School of Human Sciences (Exercise and Sport Science), The University of Western Australia, Perth, WA 6009, Australia; antonin.jamotte@research.uwa.edu.au; 3 Tennis Australia, Melbourne, VIC 3000, Australia; 4 Departamento de Desporto e Saúde, Escola de Saúde e Desenvolvimento Humano, Universidade de Évora, Largo dos Colegiais 2, 7000-645 Évora, Portugal; jparraca@uevora.pt; 5 Comprehensive Health Research Centre (CHRC), Universidade de Évora, Largo dos Colegiais 2, 7000-645 Évora, Portugal

**Keywords:** HRV, psychophysiology, performance, racquet sports

## Abstract

**Background:** This study aimed to examine changes in heart rate variability (HRV) across three match-related time points (pre-match, during the match, and post-match) and to explore whether these physiological responses differed between winners and losers in competitive padel. **Methods:** Twelve matches were analyzed, involving 11 high-level Finnish padel players ranked within the national top 24. HRV was recorded before, during, and immediately after each match, with each measurement lasting a minimum of five min. Time-domain (e.g., SDNN, RMSSD, pNN50), frequency-domain (e.g., LF, HF), and non-linear (e.g., SD1, SD2) HRV metrics were extracted for analysis. All matches took place in Tampere, Finland, under controlled conditions. **Results:** Results revealed significant intra-match fluctuations in HRV across all domains. Moreover, losing players exhibited consistently higher relative heart rate during the match, suggesting greater physiological strain. **Conclusions:** This study contributes novel evidence on the dynamic nature of autonomic responses in padel and supports the integration of HRV monitoring in performance and recovery management protocols for high-level athletes.

## 1. Introduction

Padel, a complex sport, relies on the intricate interplay of multiple factors including physiological capacities, physical attributes, psychological traits, and tactical and technical proficiency for optimal performance [1]. Throughout a standard padel match, players are engaged in continuous decision-making processes [2], interspersed with intermittent bursts of whole-body exertion, as indicated by the abundance of strokes and repeated instances of high-intensity movements like accelerations, decelerations, and directional changes [3]. Subsequent to these brief periods of anaerobic activity, players benefit from extended recovery intervals between points [4]. During these rest periods, the aerobic system plays a key role in replenishing phosphocreatine stores, clearing metabolic byproducts, and facilitating recovery from the anaerobic demands encountered during rallies.

Research delving into the physiological aspects of padel has unveiled a prevalence of moderate physiological responses [2,5], indicating a limited contribution of anaerobic glycolytic processes to energy supply [2,6]. Furthermore, studies suggest that VO_2_ does not serve as a limiting factor [6], while players often perceive a moderate to relatively high level of effort [2].

Additionally, there has been limited exploration of heart rate variability (HRV) within the context of padel [7,8,9,10]. HRV, a biomarker characterized by the fluctuation in heartbeat frequency over a specified time period, offers insights derived from the analysis of R-R intervals [11,12]. This non-invasive, painless, cost-effective, and straightforward assessment tool provides valuable information about the autonomic nervous system (ANS) function, delineating the activity of its sympathetic and parasympathetic branches [11,12,13]. A low HRV suggests a prevalence in sympathetic activity, possibly reflecting a diminished regulatory capacity of the body [14]. Consequently, lower HRV values have been recognized as indicators of fatigue and overtraining [15,16], often linked to reduced sports performance [17]. HRV thus serves as a dynamic proxy for how the body responds to internal and external demands over time. This theoretical framework positions HRV as a suitable tool for understanding how athletes’ autonomic systems react to match-related stress and competition outcomes. However, despite its proven utility in other disciplines, HRV remains underexplored in padel and lacks standardized application in this context.

Previous studies in padel have demonstrated that participating in a match significantly diminished the HRV of medium-level players [9,10], while the match result did not affect players’ HRV values. Furthermore, HRV among elite youth players was influenced by their pre-match motivation level, with matches offering a free lesson with a World Padel Tour player associated with higher mean HR, SDNN, Pnn50, and RMSSD scores [8]. Finally, HRV was higher in the first set compared to the second and third sets in elite male padel players, suggesting that there is extra stress when players are faced with overcoming a set down or have to close the match with a set in favor, in addition to the fatigue accumulated in the second set [7]. However, none of these studies have investigated whether match outcome (winning vs. losing) affects HRV in high-level adult players—an important gap in understanding the true physiological load of competition.

Therefore, the aim of this study was to investigate the changes in HRV parameters at three time points (before, during, and after a padel match) and to examine whether these physiological responses differ between winners and losers. Based on previous findings showing HRV reductions during and after match play in racquet sports, we hypothesized that HRV values would decrease from pre- to post-match, regardless of match outcome. Given the limited and inconclusive literature on outcome-based HRV differences—particularly in elite adult padel—we also explored whether losing players would exhibit greater cardiovascular strain and lower HRV values, but we treated this as an exploratory objective rather than a formal hypothesis.

## 2. Materials and Methods

### 2.1. Participants

A total of 11 high-level padel players from Finland (ranked the top 24) voluntarily participated in the present study. This represents almost 50% of the total target population. An observational study of elite athletes from volleyball included 14 athletes who were in preparation for competing in important events, representing a similar sample size in a similar context (high-level athletes prior to competition) [18]. All participants were ranked the top 24 in Finland. None of the athletes had any physical injuries nor were they taking any medication at the time of the measurements. In addition, none of the participants had any reason that prevented them from participating in the study. The study was in accordance with the Helsinki Declaration [19]. Informed consent for participation was obtained from all subjects involved in the study. Participants were treated ethically under the American Psychological Association code of ethics regarding consent, anonymity, and responses. Previously, the current investigation had been approved by the Ethics Committee of the European University of Madrid with the code CIPI/22.303.

### 2.2. Instruments and Study Variables

Heart rate and heart rate variability were assessed pre-match, during the match, and post-match, using a Polar H10 device (Kempele, Finland). This device samples data at 200 Hz, meeting the standards for short-term HRV analysis [20]. The Kubios HRV computer program (University of Kuopio, Kuopio, Finland) was used to calculate all the variables studied. The maximum, average, and minimum heart rate values were recorded and expressed as percentages of the estimated maximum heart rate. The estimated maximum heart rate was calculated with the Tanaka’s formula (208–0.7 × age) [21]. HRV values were analyzed using three methodological approaches: time-domain, frequency-domain, and non-linear analyses. Time-domain parameters included SDNN (standard deviation of normal-to-normal intervals), which reflects overall HRV; RMSSD (root mean square of successive differences), a marker of parasympathetic activity; and pNN50 (percentage of successive RR intervals differing by more than 50 ms), which also indicates vagal modulation. In the frequency domain, HF (high-frequency power) was used to assess parasympathetic activity, while LF (low-frequency power) reflects a mix of sympathetic and parasympathetic influences. Lastly, non-linear indices SD1 and SD2 provide insight into autonomic system dynamics, with SD1 primarily representing short-term variability (parasympathetic tone) and SD2 reflecting both short- and long-term HRV components.

All HRV recordings were segmented into 5 min windows, following guidelines for short-term HRV analysis [22]. Artifact correction was performed using Kubios’ automatic artifact correction algorithm (medium filter), and only segments with less than 5% correction were retained. No data transformations (e.g., logarithmic) were applied, in keeping with the exploratory nature of the study.

Match outcome was also assessed: two different groups were made according to match-winning players and match-losing players.

### 2.3. Procedure

Players were instructed to wear the Polar H10 strap. The belt was adjusted according to each participant’s chest size and tied around the chest, at the level of the xiphoid process after electrode areas were moistened by tap water. A Polar Heart Rate Monitor was then clipped onto the Polar Heart Rate Transmitter Belt of each player before. Once the researcher activated the timer, following recommendations from previous studies [23,24], participants had to be at rest for 15 min prior to baseline data collection.

Pre-match HRV was then recorded over a 5 min segment while players remained in a seated position on a chair [22]. Although the supine position is considered the gold standard for baseline HRV measurements, the seated posture was chosen to preserve ecological validity and minimize disruptions to pre-match routines. This limitation is addressed in the Discussion.

Following this, players completed their individual warm-up routines before engaging in the official pre-match warm-up, which consisted of rallying with the opponent and, as per the International Padel Federation rules, lasts five min. Subsequently, players had a two min preparation period prior to the start of the match.

When the first serve was put into play, the researcher recorded the timer’s reading. A best-of-three set match was then played, and the timer was marked again when the last point of the match concluded. The period from the first serve to the final point of the match was defined as the match time.

After the match, players were given two min to transition from the playing field to a seated position while keeping the Polar H10 strap. Once seated, they remained at rest for an additional five min [22], which was classified as the post-match period.

HRV measurements take at least five min, since it is considered the gold standard for short-term measurements [22].

### 2.4. Statistical Analysis

Descriptive statistics were presented as mean and standard deviation. Considering the results of Shapiro–Wilk and the sample size, nonparametric analyses were conducted together with the effect size. Differences between pre-match, during the match, and post-match as well as HRV variables were explored using a Friedman test, with three repeated measures (pre-, during, and post-match), together with Kendall’s W to assess effect size. To analyze pairwise comparisons, a Durbin-Conover post hoc test was used. We analyzed the effect of the match outcome (win or lose) through Mann–Whitney U tests, together with the rank-biserial correlation to assess effect sizes. The level of significance for the comparisons was set at *p* < 0.05, and Bonferroni correction was used to control Type I error. Kendall’s W effect size was interpreted as small (0.10–0.29), medium (0.30–0.50), and large (>0.50). We interpreted the rank-biserial correlation as small (0.10–0.29), medium (0.30–0.50), and large (> 0.50) [25,26,27].

All data were analyzed using the statistical package SPSS for Macintosh v. 25.0 (SPSS Inc., Chicago, IL, United States). Additionally, Jamovi 2.5.4 for Machintosh (Jamovi Project, Sydney, Australia) was used exclusively for exploratory visualizations.

## 3. Results

Table 1 shows heart rate and heart rate variability results.

**Figure 1:**
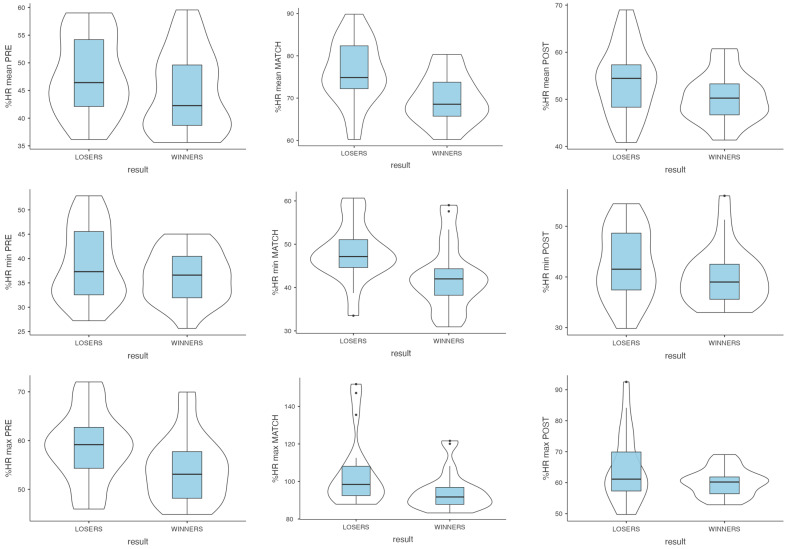

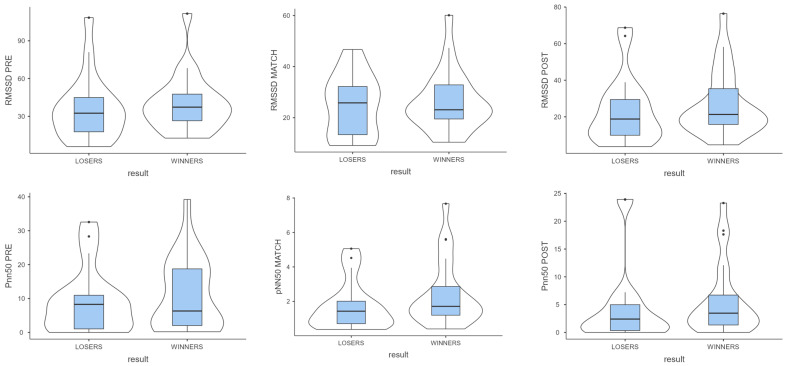
Heart rate and time-domain heart rate variability in pre-match, during match, and post-match in winners and losers.

**Figure 2:**
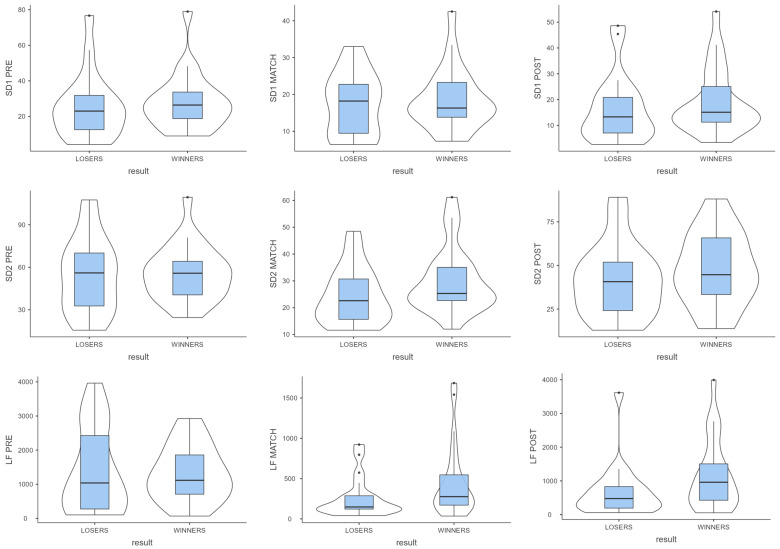

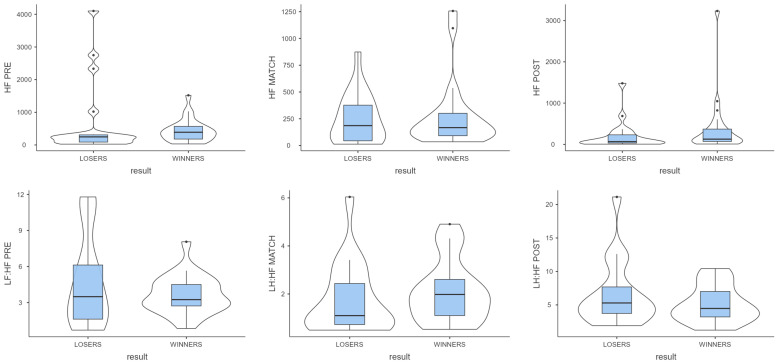
Frequency-domain and non-linear heart rate variability in pre-match, during match, and post-match in winners and losers.

Figure 1 shows heart rate and time-domain heart rate variability comparisons among pre-match, match, and post-match periods. Figure 2 shows frequency-domain and non-linear heart rate variability comparisons among pre-match, match, and post-match periods.

### 3.1. Significant Differences According to the Match Outcome

Regarding differences based on match outcome, match-losing players exhibited significantly (*p* < 0.05) higher HRmean with 144.89 bpm vs. 134.17 bpm representing 76.16% vs. 69.80% of HRmax (rank-biserial correlation = 0.558, large effect size), HRmin with 91.47 bpm vs. 81.52 bpm representing 48.08% vs. 42.45% of HRmax (rank-biserial correlation = 0.453, medium effect size), and HRmax with 199.47 bpm vs. 181.96 bpm representing 104.90% vs. 94.60% of HRmax (rank-biserial correlation = 0.428, medium effect size) during the match compared to match-winning players. Effect sizes were medium for HRmax pre (0.334), SD2 match (0.302), and LF match (0.308); small for HRmean post (0.291), HRmean pre (0.247), HRmin pre (0.149), RMSSD pre (0.169), pNN50 pre (0.135), SD1 pre (0.169), HF pre (0.254), pNN50 match (0.260), HRmean post (0.291), HRmin post (0.181), HRmax post (0.126), RMSSD post (0.199), pNN50 post (0.233), SD1 post (0.199), SD2 post (0.167), LF post (0.167), HF post (0.299), and LF/HF post (0.139); and trivial for SD2 pre (0.068), LF pre (0.080), LF/HF pre (0.038), RMSSD match (0.050), SD1 match (0.050), and HF match (0.038).

### 3.2. Significant Differences According to the Phase (Pre-Match, During the Match, Post-Match)

Regarding differences according to the phase (pre-match, during the match, post-match), there were significant differences (*p* < 0.05) in all the analyzed HR variables and in most of the analyzed HRV variables.

#### 3.2.1. HR Variables

Regarding HR variables, the significant differences (*p* < 0.05) found were the following:

Pre-match compared to during the match: HRmean with 85.5 bpm vs. 134.17 bpm for winners and 90.95 bpm vs. 144.49 bpm for losers (corresponding to 44.71% vs. 69.80% and 47.86% vs. 76.16% of HRmax, respectively), HRmin with 69.96 bpm vs. 81.52 bpm for winners and 73.89 bpm vs. 91.47 bpm for losers (corresponding to 36.41% vs. 42.45% and 38.88% vs. 48.08% of HRmax, respectively), and HRmax with 104.13 bpm vs. 181.96 bpm for winners and 111.37 bpm vs. 199.47 bpm for losers (corresponding to 54.19% vs. 94.60% and 58.57% vs. 104.90% of HRmax, respectively).

Pre-match compared to post-match: HRmean with 85.5 bpm vs. 96.96 bpm for winners and 90.95 bpm vs. 102.53 bpm for losers (corresponding to 44.71% vs. 50.41% and: 47.86% vs. 53.93% of HRmax, respectively), HRmin with 69.96 bpm vs. 77.35 bpm for winners and 73.89 bpm vs. 80.74 bpm for losers (corresponding to 36.41% vs. 40.21% and 38.88% vs. 42.46% of HRmax, respectively), and HRmax with 104.13 bpm vs. 115.78 bpm for winners and 111.37 bpm vs. 122.26 bpm for losers (corresponding to 54.19% vs. 60.19% and 58.57% vs. 64.31% of HRmax, respectively). During the match compared to post-match: HRmean with 134.17 bpm vs. 96.96 bpm for winners and 144.89 bpm vs. 102.53 bpm for losers (corresponding to 69.80% vs. 50.41%and 76.16% vs. 53.93% of HRmax, respectively), HRmin with 81.52 bpm vs. 77.35 bpm for winners and 91.47 bpm vs. 80.74 bpm for losers (corresponding to 42.45% vs. 40.21% and 48.08% vs. 42.46% of HRmax, respectively), and HRmax with 181.96 bpm vs. 115.78 bpm for winners and 199.47 bpm vs. 122.26 bpm for losers (corresponding to 94.60% vs. 60.19% and 104.90% vs. 64.31% of HRmax, respectively).

Effect sizes were large for HRmean (W = 0.554) and HRmax (W = 0.546), and small for HRmin (W = 0.258).

#### 3.2.2. HRV Variables

There were significant differences (*p* < 0.05) in most of the analyzed HRV variables pre–during–post-match.

##### Time-Domain Variables

Regarding time-domain variables (RMSSD and pNN50), the significant differences (*p* < 0.05) found were the following:

Pre-match compared to during the match: RMSSD (winners: 39.60 vs. 26.15 ms) and pNN50 (winners: 11.04 vs. 2.40%; losers: 9.11 vs. 1.72%) were higher.

Pre-match compared to post-match: RMSSD (winners: 39.60 vs. 26.86 ms; losers: 35.95 vs. 23.01 ms), and pNN50 (winners: 11.04 vs. 5.72%; losers: 9.11 vs. 4.48%) were higher.

During the match compared to post-match: pNN50 values for winners (2.40 vs. 5.72%) were lower.

Therefore, both RMSSD and pNN50 values decreased during the match and raised again during the post-match measurements but not reaching baseline measurements.

Effect sizes were small for RMSSD (W = 0.118) and pNN50 (W = 0.110).

##### Poincaré Variables

Regarding Poincaré variables (SD1 and SD2), the significant differences (*p* < 0.05) found were the following:

Pre-match compared to during the match: SD1 (winners: 28.03 vs. 18.49 ms) and SD2 (winners: 55.65 vs. 29.97 ms; losers: 54.39 vs. 24.30 ms) were higher.

Pre-match compared to post-match: SD1 (winners: 28.03 vs. 19.03 ms; losers: 25.46 vs. 16.28 ms) and SD2 (winners: 55.65 vs. 47.41 ms; losers: 54.39 vs. 41.59 ms) were higher.

During the match compared to post-match: SD2 (winners: 29.97 vs. 47.41 ms; losers: 24.30 vs. 41.59 ms) were lower.

Effect sizes were small for SD1 (W = 0.123) and medium for SD2 (W = 0.331).

##### Frequency-Domain Variables

Regarding frequency-domain variables (LF, HF, and LF/HF), the significant differences (*p* < 0.05) found were the following:

Pre-match compared to during the match: LF (winners: 1316.91 vs. 456.52 ms^2^; losers: 1366.42 vs. 264.26 ms^2^) and LF/HF (losers: 4.78 vs. 1.75) were higher.

Pre-match compared to post-match: LF (losers: 1366.42 vs. 1143.30 ms^2^), HF (losers: 670.16 vs. 210.32 ms^2^), and LF/HF (losers: 4.78 vs. 6.57 ms^2^) were higher.

During the match compared to post-match: LF (winners: 456.52 vs. 1143.30 ms^2^) and LF/HF (winners: 2.08 vs. 5.14; losers: 1.75 vs. 6.57 ms^2^) were lower.

Effect sizes were small for LF (W = 0.268), HF (W = 0.107), and LF/HF (0.260).

Taking into account all the results together, all the players increased their levels of heart rate during the match and lowered during the post-match periods, not reaching baseline periods, with cardiovascular stress after competition influence. Heart rate variability decreased during the match and raised again after the match, but not reaching baseline values, showing the autonomous nervous system stress of the competition. The losers showed increased heart rate measurements and a decreased heart rate variability measurements, including the pre-match periods, showing an increased influence of competition stress in both cardiovascular and autonomous nervous systems.

## 4. Discussion

The aim of the present research was to investigate the changes in HRV parameters at three time points (before, during, and after a padel match) and to examine whether these physiological responses differ between winners and losers. The novelty of this study stems from examining the HRV in high-level padel players discriminating between winners and losers.

The study’s hypotheses yielded mixed outcomes. The hypothesis that higher pre-match parasympathetic activity would be associated with winning outcomes was not supported, as no significant differences in HRV parameters were observed between winners and losers before the match. In contrast, the hypothesis that HRV would decrease after the match irrespective of the outcome was confirmed, with HRV values consistently lower post-match compared to pre-match. The hypothesis that winners and losers would exhibit similar normalized HRV values during the match was partially supported. While most HRV variables showed no significant differences, winners demonstrated lower HRmean, HRmin, and HRmax values, suggesting greater efficiency and reduced cardiovascular strain. Finally, the hypothesis that winners would exhibit higher HRV values post-match than losers was not supported, as no significant differences were found between the two groups in post-match HRV values.

Research has consistently demonstrated that match outcomes significantly influence the physical and physiological demands placed on players in racket sports [7]. The relationship between winning and losing appears to vary depending on the specific sport. For instance, in tennis, winners generally cover greater distances at higher speeds, suggesting that success may be linked to proactive and dynamic playing styles [28]. Conversely, in wheelchair padel and squash, losing players often record higher heart rates and cover more distance per rally, indicating greater physical strain and potentially less efficient gameplay strategies [29,30]. In the case of padel, these trends are further corroborated by findings showing that losing players experience greater physiological demands, characterized by increased heart rate and higher movement intensities [31]. Our findings align with this latter pattern: losing players exhibited significantly higher heart rate responses during match play. These observations may indicate greater match-related intensity or effort in the losing group, but the design of the current study does not allow us to establish causal explanations. It is plausible that tactical positioning, pacing strategies, or psychological stress may have contributed to these differences, but such factors were not directly assessed. Similarly to a previous study on medium-level padel players [32], our findings did not reveal significant variations in HRV between winning and losing players, regardless of whether it was before, during or after the match.

HRV has recently been shown to be an indicator of exercise threshold [33]. These results suggest that the physical loads experienced by winners and losers in matches involving the top 24 Finnish padel players are similar. Our findings further support the idea that HRV responses do not significantly differ between winners and losers, regardless of the players’ level. While previous studies have suggested that losing a match or competition can lead to heightened symptoms of depression, anxiety, and social dysfunction [34], which are also known to influence HRV [35], these effects may not have been observed in our study due to several factors. It is possible that the physical intensity and uniformity of elite match play overshadow such psychological variations. Moreover, elite players may possess better psychological coping mechanisms that buffer autonomic responses following a loss. These interpretations remain speculative and require confirmation through future studies that integrate psychological, tactical, and physiological variables. The intermittent high-intensity structure of padel seems to drive significant autonomic responses in all players, regardless of outcome. This reinforces the value of monitoring HRV to understand recovery needs and training loads in competitive contexts.

HRV has been widely recognized as a valuable tool for monitoring and managing fatigue as well as mitigating the risk of overtraining in athletes. By providing insights into the autonomic nervous system’s balance between sympathetic and parasympathetic activity, HRV serves as a non-invasive and practical indicator of an athlete’s recovery status and readiness for physical exertion [36,37]. Maintaining optimal HRV levels can help athletes and coaches adjust training loads to maximize performance while minimizing injury risk and burnout. Recent research has further highlighted HRV’s potential in tailoring individualized training programs [31], specifically to assess fatigue.

Furthermore, recent evidence suggests that physical post-exercise recovery techniques can accelerate autonomic recovery by enhancing vagally mediated HRV (vmHRV). A systematic review and meta-analysis [38] found that recovery techniques, particularly cold water immersion (CWI), had a significant positive effect on RMSSD, a key HRV marker of parasympathetic reactivation. Given the physical profile of padel, such strategies might be considered in practical applications, but their efficacy in this sport has yet to be tested.

Although not statistically significant, some trends in the data are noteworthy. Losing players showed a tendency toward higher pre-match LF/HF ratios and lower RMSSD and pNN50 values, which may reflect heightened pre-competitive stress and reduced parasympathetic modulation. This trend appeared consistent during and after the match. However, these findings should be interpreted with caution given the sample size and absence of psychological data.

Since the average ranking of the match-winning players was higher than the average ranking of the match-losing players in every single match in this study, we suggest that this prolonged stress response may reflect the psychological pressure of competing against a perceived stronger opponent, underscoring the significant influence of ranking on autonomic nervous system activity in this sport [7,8,9,10]. Players appear to be highly aware of ranking disparities before, during, and after matches, indicating that racket sports are heavily influenced by both psychological and autonomic factors. These findings suggest that perceived inferiority or fear when facing a theoretically superior opponent could pose a substantial barrier to climbing the rankings, not only due to technical–tactical limitations but also because of the psychological and physiological impact of competing against higher-ranked players [9,10]. However, we cannot draw definitive conclusions from our data. The potential impact of ranking awareness on autonomic state is a hypothesis worth exploring in future research.

### Limitations and Future Studies

This study possesses several notable strengths. Firstly, it stands among the pioneering studies that explore the HRV in padel. Secondly, it represents the first study examining HRV of high-level players, specifically distinguishing between winning and losing players.

It is crucial to acknowledge certain limitations inherent in this study. Only high-level male padel players from Finland were included. While this ensured a relatively homogeneous sample in terms of sex, training background, and competitive context, individual variability in ranking, fitness status, and age may have influenced the results. These variables were not controlled experimentally nor included as covariates, and future studies should consider incorporating them to improve internal validity and control for potential confounding effects. Additionally, anthropometric and body composition data were not collected, which may have influenced HRV between players. Future studies should include these measures to better understand their relationship with autonomic responses and match outcomes.

Moreover, although the sample size represents nearly 50% of the Finnish national top 24 players, no a priori power analysis was conducted. This limitation is common in elite sports research due to restricted access to high-performance athletes. Nonetheless, we acknowledge that the sample size may reduce the statistical power to detect subtle differences—particularly between winners and losers—and future studies with larger cohorts are recommended.

Furthermore, this study did not include traditional morning fasting-state baseline HRV measurements, as our focus was on within-match HRV fluctuations rather than long-term autonomic adaptations. While baseline HRV values can be useful in studies examining prolonged physiological trends, our approach prioritizes ecological validity, offering practical insights for real-world sports performance assessment.

Moreover, heart rate values were expressed as a percentage of maximum heart rate (%HRmax), which was estimated using the Tanaka’s formula (208–0.7 × age) [21]. Although this method is widely used in applied sports science due to its simplicity, it may introduce individual variability that could affect the accuracy of %HRmax comparisons. Future studies may consider direct HRmax assessments through maximal effort testing when feasible.

In addition, incorporating recovery interventions into future studies would provide a more comprehensive understanding of how HRV is modulated during recovery and could inform practices to optimize performance and recovery in padel.

Finally, baseline HRV was collected in a seated rather than supine position. Although this aligns with real-world pre-match conditions, future studies should consider using the supine position to follow standard physiological protocols. Also, no psychological or perceptual questionnaires were administered. Future studies should consider including measures of perceived effort, stress, or emotional state to better contextualize autonomic responses.

## 5. Conclusions

This study examined heart rate variability (HRV) responses in high-level male padel players across three match phases and compared physiological patterns between winners and losers. The results showed significant fluctuations in HRV and heart rate variables across phases (pre-match, during match, post-match), indicating the autonomic demands of competitive padel. However, no statistically significant differences in HRV were observed between winners and losers.

While match-losing players exhibited higher relative heart rate values during competition—suggesting greater cardiovascular strain—these findings should be interpreted with caution given the sample size and exploratory nature of the analysis.

Overall, the data support the use of HRV monitoring to track match-related autonomic changes in elite players. Future research should build on these results with larger samples, standardized HRV protocols, and experimental controls to better understand the interaction between physiological stress, performance, and recovery in padel.

## Figures and Tables

**Table 1:** HRV results for pre-match, during the match, and post-match in winners and losers.

	Pre		During		Post				
	Win	Lose	Win	Lose	Win	Lose	Match Effect		
	M ± SD	M ± SD	M ± SD	M ± SD	M ± SD	M ± SD	χ^2^	*p*	W
%HRmean	44.71 ± 7.33	47.86 ± 7.25	69.80 ± 5.76 *	76.16 ± 7.53	50.41 ± 5.22	53.93 ± 7.52	69.9	<0.001	0.554
%HRmin	36.41 ± 5.30	38.88 ± 7.63	42.45 ± 7.46 *	48.08 ± 6.64	40.21 ± 5.78	42.46 ± 7.08	32.6	<0.001	0.258
%HRmax	54.19 ± 6.75	58.57 ± 7.41	94.60 ± 10.30 *	104.90 ± 19.27	60.19 ± 4.44	64.31 ± 11.38	68.8	<0.001	0.546
HRmean (bpm)	85.5 ± 13.18	90.95 ± 12.39	134.17 ± 10.07 *	144.89 ± 12.81	96.96 ± 10.21	102.53 ± 12.88	69.9	<0.001	0.554
HRmin (bpm)	69.96 ± 9.70	73.89 ± 13.72	81.52 ± 13.45 *	91.47 ± 11.80	77.35 ± 11.21	80.74 ± 12.71	32.6	<0.001	0.258
HRmax (bpm)	104.13 ± 12.01	111.37 ± 12.78	181.96 ± 20.24 *	199.47 ± 34.94	115.78 ± 9.07	122.26 ± 20.17	68.8	<0.001	0.546
RMSSD (ms)	39.60 ± 22.11	35.95 ± 25.83	26.15 ± 11.75	24.52 ± 12.02	26.86 ± 17.70	23.01 ± 18.42	14.9	<0.001	0.118
pNN50 (%)	11.04 ± 10.64	9.11 ± 9.67	2.40 ± 1.88	1.72 ± 1.42	5.72 ± 6.42	4.48 ± 7.21	13.9	<0.001	0.110
SD1 (ms)	28.03 ± 15.65	25.46 ± 18.30	18.49 ± 8.31	17.33 ± 8.50	19.03 ± 12.52	16.28 ± 13.02	15.5	<0.001	0.123
SD2 (ms)	55.65 ± 19.39	54.39 ± 26.34	29.97 ± 12.06	24.30 ± 10.48	47.41 ± 20.87	41.59 ± 20.70	41.8	<0.001	0.331
LF (ms^2^)	1316.91 ± 812.00	1366.42 ± 1253.38	456.52 ± 450.66	264.26 ± 249.77	1143.30 ± 1040.944	692.74 ± 810.061	33.8	<0.001	0.268
HF (ms^2^)	436.48 ± 353.07	670.16 ± 1128.97	275.96 ± 313.00	249.79 ± 234.24	386.35 ± 677.06	210.32 ± 352.35	13.5	0.001	0.107
LF/HF	3.65 ± 1.60	4.78 ± 3.86	2.08 ± 1.29	1.75 ± 1.46	5.14 ± 2.75	6.57 ± 4.84	32.8	<0.001	0.260

Note. M: Mean. SD: Standard Deviation. %HR: Heart rate expressed as a percentage of estimated maximum heart rate (%HRmax). RMSSD (ms): Root mean square of successive RR interval differences. pNN50: Percentage of successive RR intervals that differ by more than 50 ms (%). SD1: Poincaré plot standard deviation perpendicular the line of identity (ms). SD2: Poincaré plot standard deviation along the line of identity (ms). LF: Low-frequency band (0.04–0.15 Hz) (ms^2^). HF: High-frequency band (0.15–0.4 Hz) (ms^2^). * Differences between result groups (*p* < 0.05).

## Data Availability

The raw data supporting the conclusions of this article will be made available by the authors on request.
